# Complete genome sequence of the linezolid-resistant clinical *Enterococcus faecalis* N23-3408 linked to a livestock lineage in Switzerland

**DOI:** 10.1128/mra.00249-24

**Published:** 2024-05-20

**Authors:** Magdalena Nüesch-Inderbinen, Michael Biggel, Marianne Wehrli, Andreas Hans Fischer, Roger Stephan

**Affiliations:** 1 Institute for Food Safety and Hygiene, Vetsuisse Faculty, University of Zürich, Zürich, Switzerland; 2 Central Diagnostic Laboratory, Cantonal Hospital of Schaffhausen, Schaffhausen, Switzerland; 3 Department of Medicine, Cantonal Hospital of Schaffhausen, Schaffhausen, Switzerland; Portland State University, Portland, Oregon, USA

**Keywords:** linezolid, antibiotic resistance, *E. faecalis*, urine

## Abstract

Here, we report the complete genome of human clinical linezolid-resistant *Enterococcus faecalis* N23-3408. N23-3408 harbored a 59.5 kb plasmid with antimicrobial resistance genes *cat*, *erm*(B), *fexA*, *optrA*, *tet*(L), and *tet*(M). Closely related *E. faecalis* harboring this plasmid was previously obtained from livestock animals and pet food in Switzerland.

## ANNOUNCEMENT

*E. faecalis* N23-3408 was isolated from the urine of a patient who was hospitalized with symptoms of a catheter-associated urinary tract infection (Table 1). N23-3408 was isolated from native urine using CPSE agar (bioMérieux, Petit-Lancy, Switzerland) and incubation at 37°C for 16–18 h.

**TABLE 1:** Characteristics of clinical linezolid-resistant *E. faecalis* N23-3408

Characteristics	Data
Metadata	
Strain identifier	N23-3408
Isolation source	Human urine
Sampling date	23 November 2023
Sampling location	Cantonal hospital of Schaffhausen, Switzerland
Location coordinates	47.71224, 8.636051
Illumina sequencing data	
Total read length (Mbp)	310
Total no. of reads (2×)	1,042,402
Mean coverage (*x*)	108
Oxford Nanopore sequencing data	
Total read length (Mbp)	68.1
Total no. of reads	8,647
Mean coverage (*x*)	24
N50 (kb)	19
Genome assembly	
Chromosome size (bp)	2,753,584
G + C content (%)	37.66
Chromosomal antimicrobial resistance gene	*Isa*(A)
Plasmid pN23-3408-a (bp) [rep]; antimicrobial resistance genes	(59,558) rep9a_1 [repA_N]/repUS43_1 [Rep_trans]; *cat, erm*(B), *fexA, optrA*, *tet*(L), *tet*(M)
Plasmid pN23-3408-b (bp) [rep]; antimicrobial resistance genes	(50,754) rep8b [repA_N]; –

Species identification was performed using matrix-assisted laser desorption ionization–time-of-flight (MALDI-TOF) mass spectrometry (BioMérieux, Petit-Lancy, Switzerland). Disk diffusion susceptibility testing was performed following the European Committee on Antimicrobial Susceptibility Testing (EUCAST) guidelines (1). The minimal inhibitory concentration (MIC) of linezolid was determined using a gradient strip (ETest, bioMérieux, Marcy l’Etoile, France). The isolate displayed resistance to linezolid (zone diameter 15 mm and MIC 12 mg/L, respectively), and was susceptible to amoxicillin, imipenem, ciprofloxacin, levofloxacin, nitrofurantoin, and sulfamethoxazole/trimethoprim. Notably, clinical linezolid-resistant *E. faecalis* has not been registered in the Swiss surveillance system so far (https://guide.anresis.ch/human-bacteria).

DNA was isolated from a subculture of a single colony grown on sheep blood agar at 37°C for 24 h. Genomic DNA was extracted using the MagPurix EVO 24 CE IVD Nucleic Acid Extraction System. For short-read sequencing, libraries were prepared using the Nextera DNA Flex Library Preparation Kit (Illumina) and sequenced on the Illumina MiniSeq platform (2 ×  150  bp). Read trimming and quality control were performed with fastp v0.20.1 (2). For long-read sequencing, libraries were prepared with the Rapid Barcoding Kit 24 V14 SQK-RBK114.24 kit and sequenced on a FLO-MIN114 flow cell (MinION Mk1B device, Oxford Nanopore). MinKNOW 23.11.4 was used for SUP basecalling with Dorado 7.2.13 (https://community.nanoporetech.com) and adapter trimming. Nanoq 0.9.0 (3) was used to filter long reads (PHRED score ≥10, read length ≥500 bp) and to assess their quality. A hybrid assembly was generated using the Unicycler v0.5 pipeline (4), which includes circularization and rotation of circular contigs at the *dnaA* or *repA* genes (Table 1). Resistance genes and 23S rRNA mutations were searched for with AMRFinder 3.11.20 (5) and LREFinder 1.0 (6), respectively. The sequence type (ST) was assigned using mlst 2.22.0 (7), and pairwise SNP distances determined using the CFSAN pipeline (8). Default parameters were used for all tools unless stated otherwise.

The genome of N23-3408 (ST376) consisted of a 2.7 Mbp chromosome and two plasmids (pN23-3408-a and pN23-3408-b). Details are listed in Table 1. No chromosomal mutations associated with linezolid resistance were detected. N23-3408 harbors the linezolid-resistance gene *optrA* on plasmid pN23-3408-a (Table 1). This plasmid is identical (~59.5 kb; 100% query cover, 100% sequence identity) to plasmids described in *E. faecalis* isolates recovered from livestock and pet food in Switzerland (CP116554.1, CP116558.1, CP116556.1, CP088199.1, and CP097007.1) (9–11). The genome sequences of these five isolates differed by 13–20 SNPs from N23-3408, suggesting a circulating lineage with stable plasmid maintenance. These results indicate an ongoing national spread of *E. faecalis* ST376 and its plasmid among livestock and humans in Switzerland.

## Data Availability

Sequencing data were deposited in the NCBI Sequence Read Archive (SRA) under SRR27962679 (short-read data) and SRR27962678 (long-read data) and BioProject PRJNA1076362. The assembly is available under GCA_036793375.1.
